# Adaptation of the Pivotal-Differential Genome Pattern for the Induction of Intergenomic Chromosome Recombination in Hybrids of Synthetic Amphidiploids within Triticeae Tribe

**DOI:** 10.3389/fpls.2017.01300

**Published:** 2017-07-25

**Authors:** Michal T. Kwiatek, Joanna Majka, Maciej Majka, Jolanta Belter, Halina Wisniewska

**Affiliations:** ^1^Cereal Genomics Team, Department of Genomics, Institute of Plant Genetics, Polish Academy of Sciences Poznan, Poland; ^2^Cytogenetics and Molecular Physiology of Plants Team, Department of Environmental Stress Biology, Institute of Plant Genetics, Polish Academy of Sciences Poznan, Poland

**Keywords:** allopolyploids, chromosome recombination, evolution, meiosis, mitosis, pivotal-differential theory, triticeae

## Abstract

A pivotal-differential evolution pattern is when two allopolyploids share a common genome, which is called pivotal, and differ with respect to the other genome or genomes, called differential. This feature induces the intergenomic recombination between chromosomes of differential genomes, which can lead to speciation. Our study is a cytomolecular insight into this mechanism which was adapted for the induction of intergenomic chromosome recombination in hybrids of synthetic amphidiploids *Aegilops biuncialis* × *S. cereale* (UUMMRR) and triticale (AABBRR) where R-genome was pivotal. We observed chromosome recombination events which were induced by both: (1) random chromosome fragmentation and non-homologous chromosome end joining at mitosis of root meristem cells and (2) intergenomic chromosome associations at meiosis of pollen mother cells (PMCs) of F_1_ hybrids. Reciprocal chromosome translocations were identified in six F_1_ plants and 15 plants of F_2_ generation using fluorescence *in situ* hybridization (FISH) with DNA clones (pTa-86, pTa-k374, pTa-465, pTa-535, pTa-k566, and pTa-713). We observed signals of pTa-86, pTa-535, and pTa-k566 probes in several chromosome breakpoints. The comparison of the DNA clone sequences distinguished a number of common motifs, which can be considered as characteristics of chromosome breakpoint loci. Immunodetection of synaptonemal complex proteins and genomic *in situ* hybridization analysis at meiosis of PMCs of F_1_ hybrids showed, that the homologous pairing of pivotal R—genome chromosomes is crucial for the fertility of F_1_ hybrids, however, these chromosomes can be also involved in the intergeneric recombination.

## Introduction

Intergeneric cross-hybridizations and mutations are key factors in the evolution of higher plants. Polyploidization is a specific kind of mutation, which results in the inheritance of an additional set (or sets) of chromosomes. Polyploidy is common in plants and some reports suggest that 50–70% of living plant species are polyploid (Masterson, 1994; Rieseberg and Willis, 2007) and many lineages show evidence of ancient polyploidy (paleopolyploidy) in their genomes (Otto, 2007). Polyploid plants can appear spontaneously by abnormal cell division (meiotic or mitotic failures) or by fusion of unreduced gametes, which result in chromosome set multiplication (Comai, 2005). The multiplied sets of chromosomes may originate from the same or a closely related individual (“autopolyploid”) or from the cross-hybridization of two different species (“allopolyploidy”). Both autopolyploids (e.g., potato) and allopolyploids (e.g., canola, wheat, triticale) are common among domesticated plant species.

The wheat group is an excellent example of allopolyploidization, through hybridization among species from the plant genera *Aegilops* and *Triticum*, also known as an *Aegilops*-*Triticum* complex. There are two explanations of speciation in this group. First, monophyletic evolution hypothesis is that a single wild progenitor was an ancestor of particular species or genomes. Second theory, called polyphyletic evolution, says that the wild progenitor could have been introduced into several spontaneous crosses with other species and faced with multiple events of recombination (Zohary, 1999). According to the polyphyletic hypothesis, the rate of parental genome modification in the case of evolution of polyploid species is different. In this instance, one genome is closely related or even identical to the parental one (pivotal genome), while the second—differential genome (or genomes) is much more genetically diversified (Zohary and Feldman, 1962; Feldman and Levy, 2012). For example, these two theories are adapted for explaining the B-genome origin of wheat. Polyploid wheats include two evolutionary lineages: Emmer wheats (A^u^A^u^BB) and Timopheevi wheats (A^u^A^u^GG) (Spoor, 2001) where A^u^-genome is the pivotal one. Both of them are supposed to have originated from two independent crosses involving progenitors of *Triticum urartu* Thum. ex Gandil (A^u^-genome, paternal component) and *Aegilops speltoides* Tausch (S-genome, maternal component). On the one side, it is hypothesized that B-genome is monophyletic in origin and was derived directly from *Ae. speltoides* (Maestra and Naranjo, 1998; Salse et al., 2008). On the other side, B-genome is of polyphyletic origin, and it is assumed that it has derived from more than one diploid species (Jiang and Gill, 1994). Moreover, two pivotal genomes, D and U, were identified in *Aegilops* genus, where all polyploid species were subdivided into two clusters. The D-genome cluster includes a diploid *Ae. tauschii* and six polyploid species of *Vertebrata* and *Cylindropyron* sections, while the U-genome cluster included a diploid *Ae. umbellulata* and eight polyploid species of *Pleionathera* section (Feldman, 1965). D-genome is combined with C-genome (DDCC; *Ae. cylindrica*), M-genome (DDMM; *Ae. crassa*), N-genome (DDNN, *Ae. ventricosa*), M-and S-genome (DDMMSS, *Ae. vavilovii*) and M-and U-genome (DDMMUU; *Ae. juvenalis*). Furthermore, in species of *Pleionathera* section, the U-genome is combined with C-genome (UUCC; *Ae. triuncialis*), S-genome (UUSS; *Ae. variabilis* and *Ae. kotschyi*), M-genomes (UUMM; *Ae. geniculata, Ae. biuncialis, Ae. columnaris, Ae. triaristata*) or M- and N-genome (UUMMNN, *Ae. triaristata*) (Kihara, 1954; Kilian et al., 2011).

Previous reports showed that the stabilization of allopolyploids involves: (1) chromosome diploidization, when meiotic pairing of homoeologous chromosomes is suppressed, which results in a diploid-like pairing pattern; and (2) genetic diploidization, in which duplicate genes are either silenced or expressed at reduced levels (Ozkan et al., 2001). In the case of wheat, two dominant genes, *Ph1* and *Ph2* (minor effect) are recognized to be responsible for the suppressing of homologous pairing (Riley and Chapman, 1958; Sears, 1976; Griffiths et al., 2006). Hence, during meiosis (prophase I), homologous chromosomes are coupled along their length by the loading of a protein structure, called the synaptonemal complex (SC), composed of two proteins ASY-1 and ZYP-1 (Zickler and Kleckner, 1999; Jenkins et al., 2008). Within this framework, homolog chromosomes can recombine. The expression of *Ph* genes prevents multivalent formation with its obvious consequences for uneven chromosome segregation. In other words, wheat evolved a pairing control system that enforces strictly homologous bivalent pairing.

In contrary, hybrids between related allopolyploids are common and evolved under the pivotal-differential pattern (Zohary and Feldman, 1962; Feldman, 1965; Levy and Feldman, 2002). The key feature of this type of evolution is the presence of two allopolyploids sharing a common genome or genomes and being distinguished at once by other genomes. When these two allopolyploids hybridize, the hybrid has the pivotal genomes presented twice and other genomes that differ from each other (differential genomes) at haploid stage. Such hybrids occur naturally in *A. Triticum* complex and are fertile (Kimber and Feldman, 1987). It is supposed that initial fertility in such forms is related to a buffering effect of the pivotal genome. Furthermore, rearrangements of the differential genomes subsequently occur following a recombination between them. Backcrossing of such secondary amphiploids to its parents will produce the progeny, which is phenotypically and genotypically different from the parental allotetraploids (Kimber and Yen, 1988).

Generating synthetic amphidiploids containing the genomes of different cereal species provides new insights into polyploid evolution, which can help to understand the mechanism and evolutionary aspects of polyploidy. It can also facilitate the transmission of valuable genetic properties from wild species to cultivated plants (Apolinarska et al., 2010; Kwiatek et al., 2012, 2013). Studies of synthetic allopolyploids obtained within *Brassica* or *Aegilops-Triticale* complex have revealed that polyploidization can lead to rapid and extensive genome changes (Song et al., 1995; Feldman et al., 1997; Liu et al., 1998a,b). It was reported that newly synthesized allopolyploids of wheat and *Aegilops* are characterized by the extensive genome changes at the molecular level, such as rapid elimination of specific low-copy DNA sequences (Ozkan et al., 2001; Shaked et al., 2001).

The main objective of this research was to induce the recombination events between chromosomes of *Aegilops biuncialis* and triticale subgenomes. For that purpose, we have crossed *Aegilops-Secale* amphidiploid forms (UUMMRR) with triticale (AABBRR). With this strategy, we have obtained F_1_ plants containing doubled R-genome chromosomes (pivotal genome, in this case) and complete sets of monosomic A-, B-, U-, and M-genome chromosomes. In the present work, we have karyotyped the F_1_ plants using fluorescence *in situ* hybridization (FISH), and examined the pairing interactions of chromosomes during meiosis of pollen mother cells of F_1_ plants using genomic *in situ* hybridization (GISH) and immunodetection of proteins involved in synaptonemal complex (SC) formation. Moreover, we have established the sequence patterns of certain breakpoints loci in chromosomes carrying alien chromatin translocations. Finally, the self-pollination efficiency of F_1_ plants has been calculated.

## Materials and methods

### Plant material

Seeds of two accessions of *A. biuncialis* Eig. (Ab5—14712 and Ab7—14716, respectively) were kindly provided by Prof. Moshe Feldman (Weizmann Institute of Science, Rehovot, Israel) and used for karyotyping. Amphiploid forms of *Ae. biuncialis* × *S. cereale* (Wojciechowska and Pudelska, 2002, 2005) were used for reciprocal hybridizations with hexaploid triticale (2n = 6x = 42; AABBRR; Bogo, Kitaro, Lamberto, Moreno, and Sekundo cultivars; Table 1). Crossing combinations were divided into four following groups: (1) (*Ae. biuncialis* Ab5 × *S. cereale*) × triticale (pollinator), (2) (*Ae. biuncialis* Ab7 × *S. cereale*) × triticale (pollinator), (3) triticale × (*Ae. biuncialis* Ab5 × *S. cereale*; pollinator) and (4) triticale × (*Ae. biuncialis* Ab7 × *S. cereale*; pollinator). The fertility was scored by counting the number of grains per number of pollinated flowers (Table 1). Analysis of variance (ANOVA) was conducted to determine the effect of the crossing combination on fertility. The set of F_1_ plants was germinated on a wet filter paper in the dark at room temperature for 4 days. Thereafter, the seedlings were transferred to soil for 6–8 weeks under short-day conditions (8 h light/16 h dark, 20°C/18°C). Afterwards, the plants were transferred for 6 weeks to vernalizing conditions (10 h light/14 h dark, 4°C) and returned to standard greenhouse conditions (13 h light/11 h dark, 20°C/16°C) to induce flowering. The F_2_ seeds (Table 2) were collected from the three (previously isolated) spikes after natural desiccation between July and August.

**Table 1:** Efficiency of reciprocal cross-hybridizations between *Ae. biuncialis* × *S. cereale* amphiploids and hexaploid triticale.

	**Number of pollinated flowers/number of seeds (fertility)**				
	***Ae. biuncialis*** **(Ab5)** × ***S. cereale***		***Ae. biuncialis*** **(Ab7)** × ***S. cereale***		**Total**
	**Maternal component**	**Paternal component**	**Maternal component**	**Paternal component**	
Bogo	208/32 (15.38%)	710/9 (1.26%)	148/19 (12.84%)	192/0 (0%)	1858/60 (3.23%)
Kitaro	256/11 (4.29%)	230/21 (9.13%)	188/8 (4.26%)	132/1 (0.76%)	806/41 (5.09%)
Lamberto	154/25 (16.23%)	236/3 (1.27%)	210/9 (4.29%)	152/0 (0%)	842/37 (4.39%)
Moreno	168/29 (17.26%)	354/9 (2.54%)	198/33 (16.67%)	52/16 (30.77%)	772/87 (11.27%)
Sekundo	180/20 (11.11%)	376/19 (5.05%)	166/4 (2.41%)	170/3 (1.76%)	892/46 (5.16%)
Total	966/117 (12.11%)	1906/61 (3.20%)	910/73 (8.02%)	698/20 (2.87%)	5170/271 (5.24%)

**Table 2:** Number and origin of F_2_ seeds after self-pollination of F_1_ plants.

	**Number of F**_**2**_ **seeds/number of self-pollinated F**_**1**_ **spikes (fertility)**				
	***Ae. biuncialis* Ab5 *× S. cereale***		***Ae. biuncialis* Ab7 *× S. cereale***		**Total**
	**Maternal component**	**Paternal component**	**Maternal component**	**Paternal component**	
Bogo	16/96 (0.17)	5/27 (0.19)	15/57 (0.26)	n/a	36/180 (0.20)
Kitaro	18/33 (0.55)	11/51 (0.22)	22/24 (0.92)	n/a	54/109 (0.50)
Lamberto	17/42 (0.40)	3/6 (0.50)	17/27 (0.63)	n/a	37/75 (0.49)
Moreno	9/30 (0.3)	8/27 (0.30)	16/99 (0.16)	17/48 (0.35)	50/204 (0.25)
Sekundo	16/60 (0.27)	13/57 (0.23)	8/12 (0.67)	2/9 (0.22)	39/138 (0.28)
Total	76/261 (0.29)	40/168 (0.24)	78/219 (0.36)	22/58 (0.38)	216/706 (0.31)

### Chromosome preparation for FISH/GISH

Accumulation and fixation of mitotic chromosomes were carried out according to Kwiatek et al. (2016a). The digestion was performed in 0.2% (v/v) Onozuka R-10 and Calbiochem cytohelicase (1:1 ratio) and 20% pectinase (Sigma) in 10 mM citrate buffer (pH 4.6) at 37°C for 2 h and 40 min. Metaphase chromosomes were prepared as described by Heckmann et al. (2014) with minor modifications of heating temperature reported by Kwiatek et al. (2017). Meiotic chromosomes were prepared according to Zwierzykowski et al. (2008) with modifications (Kwiatek et al., 2017).

### FISH/GISH experiments

A set of six repetitive sequences: pTa-86, pTa-k374 (28S rDNA), pTa-465, pTa-535, pTa-k566, and pTa-713 were amplified from the clones originated from BAC library of wheat published by Komuro et al. (2013). Sequences were amplified using primers designed by Kwiatek et al. (2016b) and labeled using nick translation kits according to manufacturer instructions (Jena BioScience, Jena, Germany). Probes pTa-86 and pTa-713 were labeled using digoxigenin-11-dUTP (Roche), whereas pTa-535, and pTa-k566 were labeled using tetramethyl-5-dUTP-rhodamine (Roche) and pTa-k374 and pTa-465 were labeled by Atto647 (Jena BioScience). FISH experiments were performed largely as described in Kwiatek et al. (2016b) with the following modifications. Mitotic chromosomes were denatured for 4 min at 75°C, and stringent washes were carried out in 0.1 × SSC at 42°C for 10 min. Meiotic chromosomes were denatured for 3 min at 70°C and a stringent wash was the same as above. Digoxigenin was detected by fluorescein anti-digoxigenin antibody (1:20; Roche). The post-hybridization washes were performed according to Heslop-Harrison (2000) for 30 min, at 42°C in 0,1 × SSC solution. GISH was carried out according to Kwiatek et al. (2012) with modifications (Kwiatek et al., 2016c). DNA of *Aegilops comosa* Sm. (PI 551020; U.S. National Plant Germplasm System), a progenitor of the M-genome of *Ae. biuncialis*, was labeled by nick translation with Atto-488 dye (Jena Bioscience), whereas DNA of *Ae. umbellulata* Zhuk. (U-genome progenitor; PI 222762; U.S. National Plant Germplasm System) was labeled with Atto-550 dye (Jena Bioscience). Total genomic DNA of respective triticales (Bogo, Kitaro, Moreno, Lamberto or Sekundo) was used as an unspecific blocking DNA and was sheared by boiling for 30–45 min and used at a ratio of 1:50 (probe:block). Mitotic and meiotic chromosomes were observed with an Olympus XM10 CCD camera attached to an Olympus BX 61 automatic epifluorescence microscope. Image processing was carried out using Olympus Cell-F (version 3.1; Olympus Soft Imaging Solutions GmbH, Münster, Germany) imaging software with a support of PaintShop Pro X5 software (version 15.0.0.183; Corel Corporation, Ottawa, Canada).

### Immunodetection of synaptonemal complex proteins

Meiocytes of F_1_ plants were embedded in acrylamide in order to keep their three-dimensional architecture as described by Bass et al. (1997), with the modifications (Phillips et al., 2010, 2012). Anthers at the appropriate stage of meiosis were collected into Buffer A (15 mM Pipes-NaOH, pH 6.8, 80 mM KCl, 20 mM NaCl, 0.5 mM EGTA, 2 mM EDTA, 0.15 mM spermine tetra HCL, 0.05 mM spermidine, 1 mM DTT, 0.32 M sorbitol) and fixed for 15 min in freshly prepared 4% paraformaldehyde in Buffer A. Next, anthers were macerated using a brass rod in Buffer A followed by embedding in acrylamide. The pads were incubated for overnight at 4°C in blocking buffer containing anti-ASY1 antibody raised in rabbit (Armstrong et al., 2002) and anti-ZYP1 raised in rat (Higgins et al., 2005) diluted 1:200 and 1:250, respectively. Then, the pads were washed in PBS + 0.1% Tween 20 + 1 mM EDTA pH 8 at room temperature followed by fixation in 2% paraformaldehyde in Buffer A and washed in PBS + 0.1% Tween 20 + 1 mM EDTA pH 8 at room temperature. Finally, pads were mounted in mounting medium (200 mM Tris-HCl pH 8, 2.5% DABCO (1,4-diazobicyclo(2,2,2)octane; Sigma), 80% glycerol and 1 mg/ml DAPI. Nuclei were examined and optically sectioned using a Leica TCS SP5II confocal laser scanning microscope supplied with Leica LAS-AF software.

### Comparison of breakpoint associated repetitive sequences

Sequences of following BAC clones: pTa-86, pTa-535, and pTa-k566 (KC290896, KC290894, and KC290904, respectively; NCBI Genebank), which were mapped in the chromosome breakpoints were compared using Standard Nucleotide Basic Local Alignment Search Tool (Altschul et al., 1990, 1997). The comparison was performed using BLASTn program (NCBI, 2017) to work out the similar sequence motifs.

## Results

### Production of (*Ae. biuncialis* × *S. cereale*) × *triticale* F_1_ and F_2_ seeds

In the first step, F_1_ hybrids were developed by crossing *Aegilops*-*Secale* amphidiploid forms (UUMMRR) with triticale (AABBRR) to induce the recombination events between chromosomes of *Aegilops* and triticale genomes, assuming that R-genome is the pivotal one. The reciprocal hybridizations involved 5170 flowers (pollinated manually), which yielded 271 F_1_ seeds and the fertility was 5.24% (Table 1).The null hypothesis stated that the means of fertility scores of crossing combinations were equal. Because the *p*-value was 0.3818, which was greater than the significance level of 0.05, there was not enough evidence to reject the null hypothesis. However, after comparing the overall scores, the fertility was higher, when the *A. Secale* amphiploids were used as maternal components. F_2_ seeds (216) were obtained by the self-pollination of spikes (706) of F_1_ plants, which means that every third self-pollinated spike delivered a F_2_ seed (Table 2).

### Karyotyping of *Ae. biuncialis* chromosomes

**Figure 1:**
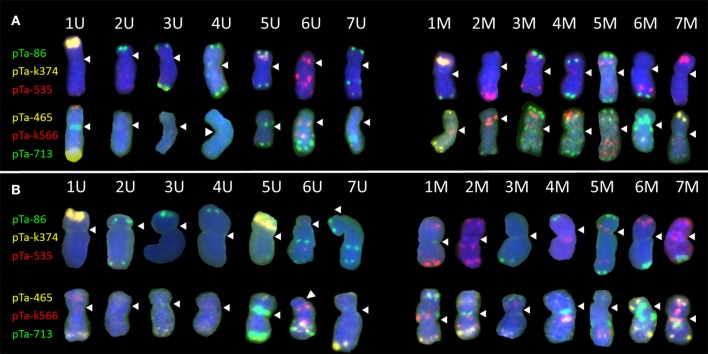
Karyograms of *Aegilops biuncialis* **(A)** Ab5–14712 and **(B)** Ab7–14716, showing U- and M-genome chromosomes after FISH with two sets of probes. First set: pTa-86 (green), pTa-k374 (yellow), and pTa-535 (red); second set: pTa-465 (yellow), pTa-k566 (red), and pTa-713 (green). Spearheads indicate centromere locations.

The FISH technique was applied to mitotic chromosomes of *Ae. biuncialis* Ab5 (Figure 1A) and Ab7 (Figure 1B), which were used for *Ae. biuncialis* × *S. cereale* amphiploids production, followed by reciprocal hybridizations with triticale cultivars. Karyotyping was performed by the signal patterns comparison according to a previous study (Kwiatek et al., 2013) and analogous cytogenetic studies (Schneider et al., 2005; Komuro et al., 2013) in purpose to track *Aegilops* chromosomes in subsequent generations of (*Ae. biuncialis* × *S. cereale*) × triticale hybrids. Moreover, GISH analysis was used to discriminate the U- and M-genome, as well.

Most of the U-genome and M-genome chromosomes showed similarities of hybridization patterns, comparing Ab5 and Ab7 karyotypes, but some differences were also observed. In both of the *Ae. biuncialis* accessions analyzed, the pTa-86 probe hybridized mostly to the telomeric region of each chromosome. However, on chromosome 6 UL (Ab 7) and 7 UL (of Ab5 and Ab7), an additional interstitial site was observed. Probe pTa-k374, which indicates 35S rDNA loci, gave strong signals on 1 U chromosomes of both accessions and on 1 M chromosomes of Ab 5. Similarly, pTa-k374 was stronger on chromosome 5 U of Ab7 accession, when compared to Ab5Probe pTa-535 was more abundant on M-genome chromosomes. Comparing both accessions, polymorphisms between pTa-535 signal localization were observed on 6 U, 6 M, and 7 M chromosomes. Signals of pTa-465 were observed mainly in the distal regions of chromosomes. We observed the absence of pTa-465 signals on 6 U and 6 M chromosomes of Ab5 in comparison to Ab7. Signals of pTa-k566 probe were localized in the interstitial regions of 6 U, 1 M, 2 M, 3 M, 4 M, 5 M, and 7 M and in the telomeric regions of 1 U and 4 M. Comparing both accessions, differences in localization of pTa-k566 probe signals were revealed on chromosomes 2 M. Hybridization patterns of pTa-713 probe were the same for 5 U, 6 U, 6 M, and 7 M, however on chromosomes 1 U, 7 U, 2 M, and 5 M interspecific differences were observed. Moreover, this probe did not perform any signals on 2 U, 3 U, and 4 U chromosomes.

### Chromosome identification of (*Ae. biuncialis* × *S. cereale*) × *triticale* F_1_ and F_2_ hybrids

**Figure 2:**
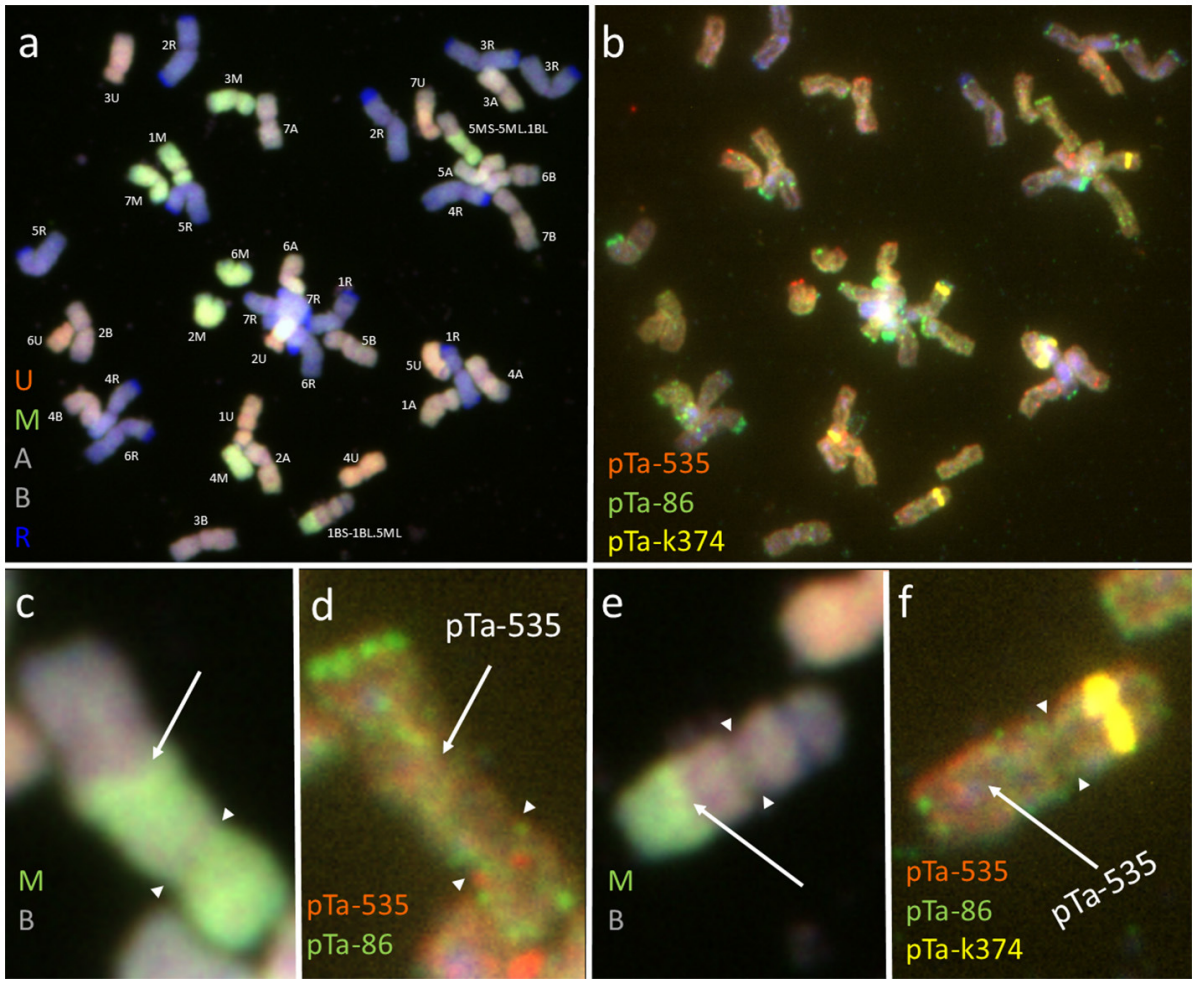
Mitotic chromosomes of (*Ae. biuncialis* Ab7 × *S. cereale*) × Sekundo F_1_ hybrid after **(a,c,e)** GISH with genomic DNA probes of: *Ae. umbellulata* (UU; red), *Aegilops comosa* (MM; green) blocked with genomic DNA of triticale “Sekundo” (A- and B-genome chromosomes—gray, R-genome chromosomes—blue); **(b,d,f)** FISH with pTa-86 (green), pTa-k374 (yellow), and pTa-535 (red) probes. **(c,d)** 5MS-5ML.1BL chromosome translocation. **(e,f)** 1BS-1BL.5ML chromosome translocation. Spearheads indicate the centromere location. Arrows show the chromosome breakpoint localization and associated probe signal.

**Figure 3:**
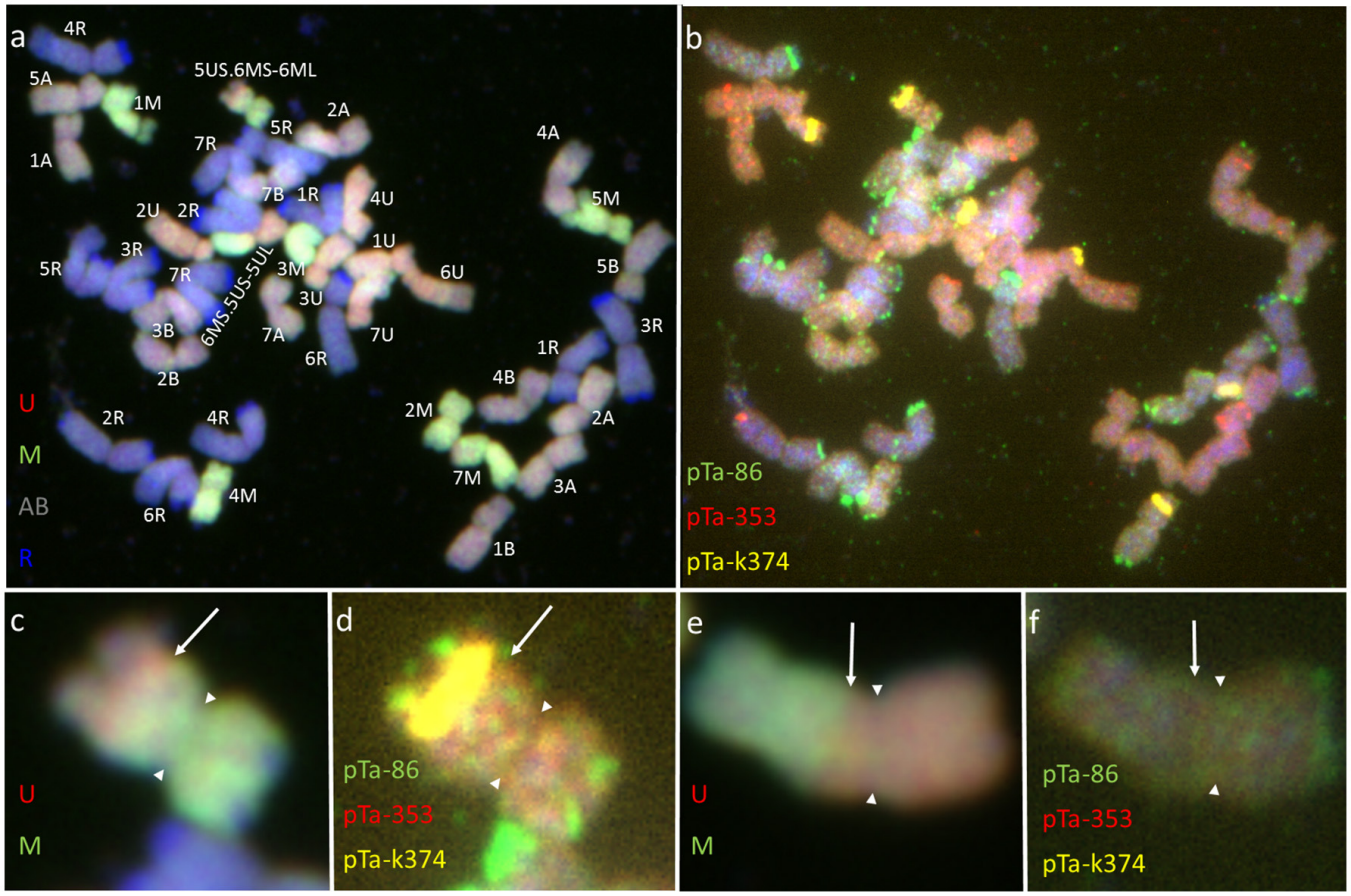
Mitotic chromosomes of Kitaro × (*Ae. biuncialis* Ab5 × *S. cereale*) F_1_ hybrid after **(a,c,e)** GISH with genomic DNA probes of: *Ae. umbellulata* (UU; red), *Aegilops comosa* (MM; green) blocked with genomic DNA of triticale “Sekundo” (A- and B-genome chromosomes—gray, R-genome chromosomes—blue); **(b,d,f)** FISH with pTa-86 (green), pTa-k374 (yellow), and pTa-535 (red) probes. **(c,d)** 5US.6MS-6ML chromosome translocation. **(e,f)** 6MS.5US-5UL chromosome translocation. Arrows show the chromosome breakpoint localization. Spearheads indicate the centromere location.

The mitotic chromosomes of 236 F_1_ generation plants (Table 3) were distinguished using FISH and genome-assigned using GISH. Ten cells per plant were analyzed, which made 2,360 examined cells, in total. As expected, the chromosome constitution of F_1_ plants consisted of 14 R-genome chromosomes and 7 A-, 7 B-, 7 U-, and 7 M-genome chromosomes. The total number of chromosomes was 42. However, six plants were detected to carry chromosome translocations (Table 4). Moreover, particular chromosomes involved in those translocation events varied within a plant, to be more precisely—root meristem, which resulted in differences of chromosome constitution between cells of the same meristem were observed. The chromosome translocations encompassed both intergeneric (*Aegilops*-*triticale*) and intrageneric (*Aegilops-Aegilops*) recombination (Figures 2, 3, respectively). In particular, following chromosomes of triticale: 1 B, 4 R, 5 R, 7 R, and *Aegilops*: 4 U, 5 U, 7 U, 5 M, 6 M, and 7 M were involved in recombination events (Table 4). What is more, the chromosome breakpoint regions overlapped with three FISH probe loci (pTa-86, pTa-535, and pTa-k566; Figures 2d,f). The FISH/GISH analyses of F_2_ hybrids were performed on 216 plants (2,160 cells, as a consequence). Translocation chromosomes were identified in 15 plants (Table 4). The chromosome number of F_2_ plants ranged from 35 to 41. As in F_1_ plants, the same chromosomes were involved in translocation events, with the exception of 7RS.3AS-3AL translocation chromosome, which was identified only in two plants of F_2_ generation. In the rest of the cases, chromosome 5 M of *Ae. biuncialis* was involved in intergeneric chromosome recombination with 5 U, 5 R, and 1 B chromosomes. In contrast to the chromosome aberrations observed in F_1_ plants, all cells of particular root meristem carried the same chromosome set and structure.

**Table 3:** Number and origin of F_1_ plants karyotyped in the study.

	***Ae. biuncialis* (Ab5) *× S. cereale***		***Ae. biuncialis* (Ab7) *× S. cereale***		**Total**
	**Maternal component**	**Paternal component**	**Maternal component**	**Paternal component**	
Bogo	19	0	32	9	60
Kitaro	8	1	11	17	37
Lamberto	9	0	14	2	25
Moreno	33	16	10	9	68
Sekundo	4	3	20	19	46
Total	73	20	87	56	236

**Table 4:** Chromosome translocations identified in root meristems of F_1_ and F_2_ (*Ae. biuncialis* × *S. cereale*) × triticale hybrids.

**Crossing combination**	**Number of plants**	**2n**	**Number of cells with (and without) chromosome translocations**	**Types of chromosome translocations (probe signal mapped in chromosome breakpoint loci)**
**PLANTS OF F**_1_ **GENERATION**				
(*Ae. biuncialis* Ab7 *× S. cereale*) × Sekundo	1	42	2 (8)	1BS-1BL.5ML and 5MS-5ML.1BL (pTa-86) or 7RS-7RL.7UL and 7US-7UL.7RL (pTa-535)
(*Ae. biuncialis* Ab5 *× S. cereale*) × Moreno	1	42	2 (8)	5RS-5RL.5ML and 5MS-5ML.5RL (pTa-k566) or 4RS-4RL.4UL and 4US-4UL-4RL (none)
(*Ae. biuncialis* Ab5 *× S. cereale*) × Moreno	1	42	1 (9)	5US.2RS-2RL (pTa-535)
(*Ae. biuncialis* Ab7 *× S. cereale*) × Moreno	1	42	1 (9)	7US.6BS-6BL and 6BS.7US-7UL (pTa-86)
(*Ae. biuncialis* Ab7 *× S. cereale*) × Moreno	1	42	1 (9)	7US-7UL.7ML and 7MS-7ML-7UL (pTa-535)
Kitaro × (*Ae. biuncialis* Ab5 *× S. cereale*)	1	42	1 (9)	5US.6MS-6ML and 6MS.5US-5UL (none)
**PLANTS OF F**_2_ **GENERATION**				
(*Ae. biuncialis* Ab5 *× S. cereale*) × Moreno	2	35; 37	10 (0)	5MS.5US-5UL (pTa-535)
(*Ae. biuncialis* Ab5 *× S. cereale*) × Moreno	1	35	10 (0)	5US.5MS-5ML (pTa-535)
(*Ae. biuncialis* Ab5 *× S. cereale*) × Moreno	2	36; 39	10 (0)	5RS-5RL.5ML (pTa-535)
(*Ae. biuncialis* Ab5 *× S. cereale*) × Sekundo	3	33; 37; 39	10 (0)	1BS-1BL.5ML (pTa-86)
(*Ae. biuncialis* Ab7 *× S. cereale*) × Moreno	2	35; 39	10 (0)	1BS-1BL.5ML (pTa-535)
(*Ae. biuncialis* Ab7 *× S. cereale*) × Moreno	3	35; 39; 41	10 (0)	5MS-5ML.6BL (pTa-86)
Kitaro × (*Ae. biuncialis* Ab5 *× S. cereale*)	2	39; 41	10 (0)	7RS.3AS-3AL (none)

### Comparison of fish probe sequences mapped in chromosome breakpoint loci

DNA repetitive sequences of four BAC clones: pTa-86 (KC290896), pTa-535 (KC290894), and pTa-k566 (KC290904) were compared using the BLASTn software. The aim of this approach was to find and dissect DNA motifs which are similar between each BAC sequence. All comparisons resulted in a number of identical or akin motifs (Table 5). The sequences of pTa-86 and pTa-535 clones were most related and shared 20 common DNA motifs, 8–14 nucleotides long. Nevertheless, only five motifs were concerted for pTa-86 and pTa-k566 clone sequences. Neither of those DNA motifs was presented in all BAC clone sequences. The most common motifs consisted of eight bases and were similar to TCAATTTC or TCGGTCAT or TTGACCAAT sequences, considering alternations of certain bases and their order changes.

**Table 5:** Common motifs shared by DNA repetitive sequences of BAC clones mapped in chromosome breakpoints in FISH experiment.

	**pTa-86**	**pTa-535**	**pTa-k566**
pTa-86	x	GTTTGTCCgATT TGgAGTGATTTcCA GTTTGTCCgATT GTCATCAAT GTTCAAAA TCATCAAT TCAATTTC TCGGTCAT TCAATTTC AGTGATTTcCA AATTCTGA TGTTGAAcCTT CATTTCAT AACCCTGA GTCATCAA GTCATCAA TCAATTTC TCGGTCAT AATTCTGA TGTTGAAcCTT	CAGCACTCGC GTGGACTA GTGGACTA TACTCACC TACTCACC
pTa-535	TGaAAATCACTaCA GTCATCAAT AATtGGACAAAC AATtGGACAAAC TTTTGAAC TGaAAATCACT GTCATCAA TCAATTTC GAAATTGA TCAATTTC CATTTCAT GTCATCAA AATTCTGA AATTCTGA TCAGGGTT TCGGTCAT TCGGTCAT TCATCAAT TGTTGAAaCTT TGTTGAAaCTT	x	TTTTTtAAACTTA TTTTTtAAACTTA TTTTTtAAACTTA TTTTTtAAACTTA GAGGGTCAC TTTTtAAACTTA TTGAAAATCaCTAC TGAAAATCC TTTTtAAACTTA TTGAAAATcACTAC TTGAAAATCaCTAC TGAAAATCC GTGCGAAA
pTa-k566	CAGCACTCGC TAGTCCAC TACTCACC TAGTCCAC TACTCACC	TAAGTTTgAAAAA TAAGTTTgAAAAA TAAGTTTgAAAAA TAAGTTTgAAAAA TTGAAAATC-CTAC TTGAAAAT-ACTAC TTGAAAATC-CTAC GTGACCCTC TAAGTTTgAAAA TAAGTTTgAAAA TGAAAATCC TGAAAATCC GTGCGAAA	x

### Immunolocalization of synaptonemal complex proteins at prophase I of meiosis of pollen mother cells (PMCs) of F_1_ hybrids

**Figure 4:**
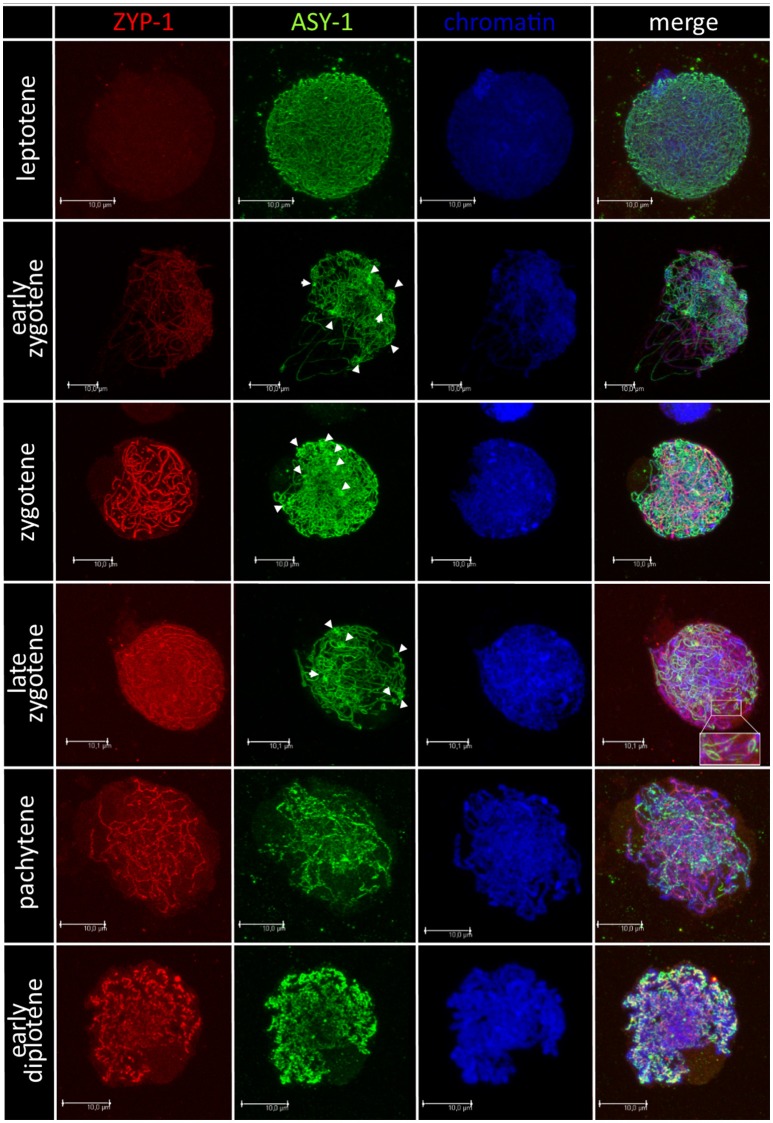
Immunodetection of ASY-1 (green) and ZYP-1 (red) foci associated with chromatin (blue) of (*Ae. biuncialis* Ab7 × *S. cereale*) × Sekundo F_1_ hybrid during subsequent stages of prophase I of meiosis of pollen mother cells. Spearheads show highly condensed zones of ASY-1 protein foci.

Chromosome pairing analyses were performed for 28 F_1_ hybrids, in particular, for 15 plants carrying *Ae. biuncialis* (Ab5) chromosomes and for 13 plants with *Ae. biuncialis* (Ab7) chromosomes. Ten PMCs per plant were examined (280, in total). In purpose to track the temporal and spatial expression of the synaptonemal complex, meiocytes were embedded in polyacrylamide gel and two structural proteins of SC (ASY1 and ZYP1) were detected using immunolocalization (Figure 4). At the leptotene, ASY1 proteins formed highly muddled, long and thin linear signals. While the meiotic process proceeds to early zygotene, the ZYP1 linear signals appeared but not co-localized with ASY1 signals. Moreover, it was observed that ASY1 proteins created knot-like, highly condensed zones. At zygotene, the linear signals of ASY1 and ZYP1 thickened similarly with the chromosome condensation. At late zygotene ASY1 loops were visible. When the chromosomes became short and thick like at the pachytene stage, the ZYP1 signals were massive, while the ASY1 signals became wavy. As the diplotene stage began, the signals of both proteins (ASY1 and ZYP1) were thick and curly and in some zones colocalized together. When the diplotene proceeded, the ZYP1 signal disappeared and the ASY1 signal became highly condensed.

### Evaluation of chromosome pairing at metaphase I of meiosis of pollen mother cells (PMCs) of F_1_ hybrids

**Figure 5:**
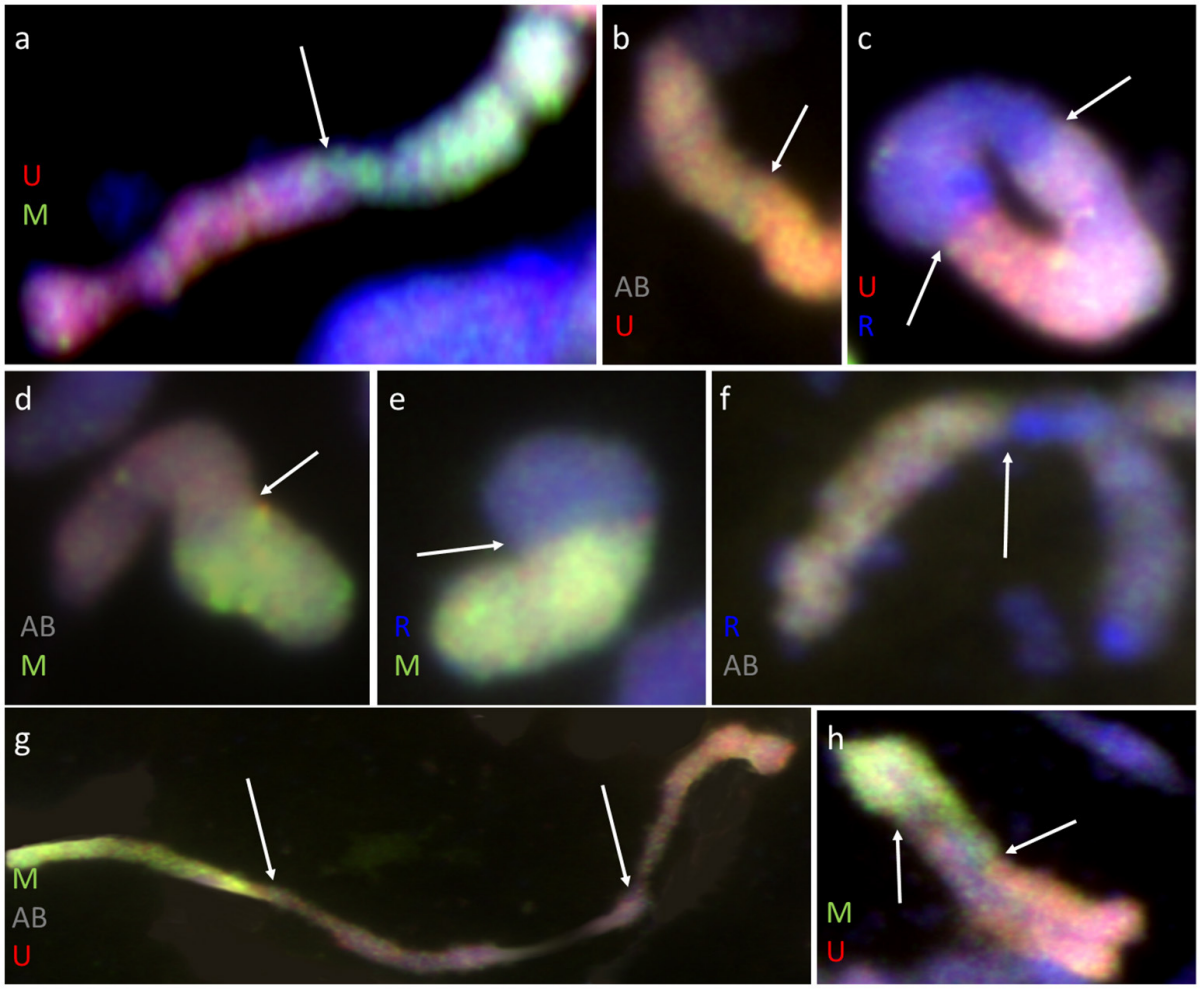
Meiotic chromosomes of (*Ae. biuncialis* Ab7 × *S. cereale*) × Sekundo F_1_ hybrid after GISH with genomic DNA probes of: *Ae. umbellulata* (UU; red) and *Ae. comosa* (MM; green) blocked with genomic DNA of triticale “Sekundo” (A- and B-genome chromosomes—gray, R-genome chromosomes—blue). Examples of seven types of intergenomic MI associations: **(a)** U-/M-genome rod bivalent; **(b)** T(A- or B-genome)/U-genome rod bivalent; **(c)** R-/U-genome ring bivalent; **(d)** T(A- or B-genome)/M-genome rod bivalent; **(e)** R-/M-genome rod bivalent; **(f)** T(A- or B-genome)/R-genome rod bivalent; **(g)** M-/T(A- or B-genome)/U-genome rod trivalent; **(h)** U-/M-/U-genome V-shape trivalent. Arrows indicate intergenomic chromosome pairing events.

Furthermore, pairing between triticale and *Ae. biuncialis* chromosomes was detected by means of genomic *in situ* hybridization in metaphase I of meiosis in the F_1_ hybrid plants. Seven univalents of each of U, M, A, and B genomes were observed in most of PMCs. Moreover, in the majority of cells, the R-genome chromosomes were paired and formed 7 bivalents. However, the intergenomic chromosome pairing was uncovered as well (Table 6, Figure 5). The GISH experiments showed that the homeologous recombination between chromosomes of different genomes was present in the form of bivalents (Figures 5a–f) and trivalents (Figures 5g,h). Moreover, both intraspecific and interspecific recombination events were detected. The ANOVA calculations followed by Tukey's honest significant difference test showed that in some cases the differences between the number of intergeneric chromosome recombination events was significant, which corresponded with the accession of *Ae. biuncialis* or/and cross-combination type. For example, the intragenomic pairing frequency between U- and M-genomes (Figure 5a) was significantly higher (α = 0.01), while comparing (*Ae. biuncialis* Ab5 × *S. cereale*) × triticale and (*Ae. biuncialis* Ab7 × *S. cereale*) × triticale hybrids. Bivalents and trivalents consisted of *Ae. biuncialis* and triticale chromosomes were observed as well. Considering the recombination events between M- and R-genome chromosomes (Figure 5E), it was showed that there were significant differences between bivalent frequencies which were correlated with the type of cross-combination. In this particular case, the M-/R-genome bivalents were observed only in triticale × (*Ae. biuncialis* × *S. cereale*) F_1_ hybrids (amphiploid form as a maternal component). The same correlation was observed for M-/T(A- or B-genome) bivalents formation (Figure 5d) which significantly differed (α = 0.01), considering the origin of the maternal component of the crossing combination. What was interesting, the highest significant (α = 0.05) differences between the means of bivalents, composed of U-genome paired with triticale chromosomes (A-, B- or R-genome), were related to both types of crossing combination and the origin of an amphiploid form (Figures 5b,c). The pairing events within triticale chromosomes, in particular between R-genome and A- or B-genome chromosomes, were also observed (Figure 5f), but did not differ in terms of cross-hybridization types. Meiotic chromosomes of *Ae. biuncialis* genomes were also associated in trivalents and appeared in all of the crossing combinations. The tendency for U-/M-/U-genome trivalents (Figure 5h) formation was significantly higher (α = 0.01) in triticale × (*Ae. biuncialis* Ab7 × *S. cereale*) combination. Remarkably, only one type of trivalent, the one containing chromosomes of both triticale and *Ae. biuncialis* (U, M, A or B genomes) was identified in (*Ae. biuncialis* Ab7 × *S. cereale*) × triticale combination (Figure 5g).

**Table 6:** Intergenomic chromosome associations during meiosis of pollen mother cells in F_1_ hybrids.

**Crossing combinations**	**Number of plants/number of PMCs**	**Number of bivalents—means (range)**						**Number of trivalents means (range)**	
		**U/M**	**U/T**	**U/R**	**M/T**	**M/R**	**T/R**	**U/M/U**	**U/T/M**
1. (*Ae. biuncialis* Ab5*× S. cereale*) × triticale	7/70	0.34 (0–1)	0.14 (0–1)	0.21 (0–1)	0.09 (0–1)	0.24 (0–1)	0.1 (0–1)	0.1 (0–1)	0 (0)
2. (*Ae. biuncialis* Ab7 *× S. cereale*) × triticale	6/90	0.13 (0–1)	0.2 (0–1)	0.15 (0–1)	0.27 (0–1)	0.33 (0–1)	0.17 (0–1)	0.17 (0–1)	0.17 (0–1)
3. Triticale × (*Ae. biuncialis* Ab5*× S. cereale*)	8/80	0.09 (0–1)	0.28 (0–1)	0.09 (0–1)	0.44 (0–2)	0 (0)	0.1 (0–1)	0.125 (0–1)	0 (0)
4. Triticale × (*Ae. biuncialis* Ab7 *× S. cereale*)	7/70	0.2 (0–1)	0.37 (0–1)	0 (0)	0.29 (0–1)	0 (0)	0.14 (0–1)	0.38 (0–1)	0 (0)
ANOVA	*F*-value	6.11	3.71	6.22	6.54	20.73	0.67	8.32	14.46
	*P*-value	0.000489	0.012104	0.000422	0.000275	<0.0001	0.571093	<0.0001	<0.0001
Tukey's honest significant difference (HSD)	HSD_0.05_	0.17	0.19	0.13	0.21	0.14	n/a	0.17	0.08
	HSD_0.01_	0.2	0.23	0.16	0.26	0.16	n/a	0.2	0.09
1. vs. 2.		*P* < 0.01	n/s	n/s	n/s	n/s	n/a	n/s	*P* < 0.01
1. vs. 3.		*P* < 0.01	n/s	n/s	*P* < 0.01	*P* < 0.01	n/a	n/s	n/s
1. vs. 4.		n/s	*P* < 0.05	*P* < 0.01	n/s	*P* < 0.01	n/a	*P* < 0.01	n/s
2. vs. 3.		n/s	n/s	n/s	n/s	*P* < 0.01	n/a	n/s	*P* < 0.01
2. vs. 4.		n/s	n/s	*P* < 0.05	n/s	*P* < 0.01	n/a	*P* < 0.01	*P* < 0.01
3. vs. 4.		n/s	n/s	n/s	n/s	n/s	n/a	*P* < 0.01	n/s

*U (Ae. biuncialis), M (Ae. biuncialis), R (S. cereale)—genome symbols. T- represents A- and B-genome of triticale, jointly. n/s, Non significant, P-value was greater than the significance level (both α = 0.01 and α = 0.05), hence there was not enough evidence to reject the null hypothesis, which stated that the means of bivalent number are equal. n/a, Not applicable*.

## Discussion

In general, F_1_ hybrids obtained by the intergeneric cross-hybridization are sterile, mostly because of the lack of functional gametes. This is connected with the differences in chromosome origin and number. If parents are of distant genome affinity and differing chromosome pair number, the F_1_ offspring will be unable to produce chromosomally identical and balanced gametes. However, especially in the evolution of the majority of polyploid plants, those obstacles were overcame with the pivotal-differential origin pattern. In this study, a pivotal-differential evolution mechanism was adapted for the induction of recombination events between chromosomes of different genomes of artificial allopolyploids from *A. Triticum* complex. Hence, we have used amphiploid forms which carry whole *Ae. biuncialis* (UUMM) and *S. cereale* (RR) chromosome sets for reciprocal cross-hybridizations with triticale (AABBRR), which is an artificial cereal, composed of A- and B-genomes of wheat and R-genome of rye (*S. cereale* L). The genome composition of F_1_ offspring which carried R-genome chromosomes present twice (pivotal or axial genome) and A-, B-, U-, and M-genome chromosomes occurring in haploid stage, allowed to produce semi-fertile gametes.

The main assumption of this work was to explore the process of the intergenomic chromosome translocations being formed during the pivotal-differential evolutionary pattern. The reciprocal cross-hybridization efficiency between *A. Secale* amphiploids and triticale was determined by the parental components, however the differences between particular crossing combinations were not significant. Nevertheless, the fertility of F_1_ plants, which was measured by the seed number per spike characteristic, was similar for all F_1_ crossing combinations. Hence, it can be said that the presence of pivotal, R-genome chromosomes secured the semi-fertility of F_1_ plants which were able to produce offspring. This statement was proven by the results of evaluation of the synaptonemal complex formation and chromosome pairing at prophase I and metaphase I of meiosis, respectively. In order to track the progress of synapsis, two proteins of synaptonemal complex, ASY1 and ZYP1, were detected and tracked using immunolocalization in meiocytes at subsequent stages of prophase I of meiosis of PMCs. The SC-associated protein ASY1 marked unpaired axial elements and the transverse filament protein ZYP1 labeled synapsed regions. In diploids, the ASY1 signal disappears in synapsed regions at zygotene and appears again at pachytene, which is supposed to be related to a temporary masking or modification of its epitope or alternatively, the protein is removed from the chromosome axes during zygotene and reloaded during pachytene (Phillips et al., 2012). In our study, the presence of both proteins at zygotene was detected in all PMCs of F_1_ hybrids, which showed that only a part of pre-condensed chromosomes were able to form synapsis and be paired, as a consequence. Furthermore, ASY1 protein appeared more condensed in several foci at zygotene and early pachytene, and created a knot-like structures. What is interesting, associated regions of chromatin in nuclei have a lower density of DAPI staining, which is characteristic for euchromatin. The same observations were reported by Phillips et al. (2012), who tracked the behavior of telomeres using FISH associated with the immunolocalisation of ASY1 protein and speculated that the co-localization of euchromatin and ASY1 at this stage may be functionally related, and might be connected with the synapsis of telomeres in these areas. In our study, the ASY-1 knot-like foci might indicate the pivotal, R-genome telomere synapsis. However, it is also possible that ASY1 loops are, in fact, in the same region.

The formation of seven R-genome bivalents was observed at metaphase I of PMCs using GISH. However, other chromosome associations were detected, as well. In theory, the presence of two homologs of pivotal, R-genome chromosomes should result in R-genome bivalent formation and lower tendency to create intergenomic chromosome pairing. These conditions should determine the A-, B-, U-, and M-genome univalent presence or intergenomic chromosome associations within those four, differential genomes. However, in our work we observed that in some cases the mean number of U/R or M/R bivalents was comparable or even higher than the mean number of U/T or M/T bivalents. It can be suspected, that *Ph1* gene (5 B chromosome) expression, which ensures the recombination only between pairs of homologous chromosomes or/and hampers pairing events between chromosomes from the related (homoeologous) sub-genomes (Griffiths et al., 2006) suppressed the formation of U/T and M/T chromosome associations. For example, Lukaszewski and Kopecky (2010) studied autotetraploid rye with additional chromosomes of wheat and reported that *Ph1* gene operates in rye in the same way, like in polyploid wheats and have suggested that it controls a basic mechanism of chromosome recognition (Lukaszewski and Kopecky, 2010). This is the most probable explanation of relatively low appearance of intergenomic chromosome recombination during meiosis of F_1_ hybrids. Moreover, the means of bivalent and trivalent number of particular chromosome associations differed significantly with respect to the parental components. Based on this fact, it can be said that the tendency to form intergenomic chromosome recombination events could depend on the origin of *Ae. biuncialis* chromosomes. To be more precise, FISH analysis was performed for the chromosome identification. At the beginning, we karyotyped two *Ae. biuncialis* accessions (Ab5 and Ab7) which were used for *A. Secale* amphiploid production (Wojciechowska and Pudelska, 2002, 2005). As expected, M-genome chromosomes showed more differences between the accessions in comparison to U-genome chromosomes, which is in parallel with the pivotal-differental composition of this species (Zohary and Feldman, 1962). Moreover, chromosome 4 M was most polymorphic, which was also reported by Schneider et al. (2005).

Six repetitive DNA sequences used as FISH probes allowed to recognize all chromosomes and segments of translocation chromosomes. Three of them (pTa-86, pTa-535, and pTa-k566) were mapped in chromosome breakpoints, that is in line with the previous reports, showing that chromosome breakpoints appear in regions which are rich in repetitive DNA sequences. The sequences comparison led us to the assumption, that 8-bases motifs, such as TCAATTTC, TCGGTCAT, TTGACCAAT or familiar are characteristic for heterochromatin regions, where the chromosome break formation takes place. Repetitive DNA sequences are able to mobilize through the genome and to change their copy number. Hence, it is hypothesized that these repetitive DNA sequences may promote chromosomal rearrangements. It was reported that satellite DNA families are involved in recombination events in *Drosophila* (Kuhn et al., 2009) and play an important role in evolution of chromosomes in mammalians (Adega et al., 2009) and plants (Raskina et al., 2008). It was also reported that mini- and microsatellite sequences can be associated with recombination hot spots in humans (Majewski and Ott, 2000) and with chromosomal fragile sites in eukaryotes (Ruiz-Herrera et al., 2006). In plants, an association between chromosome regions carrying simple sequence repeats and intergenomic translocation breakpoints in natural populations of allopolyploid wild wheats was reported, as well (Molnar et al., 2011).

Given the structure of the chromosome breakpoints, we studied the mechanism of the chromosome segments translocation, as well. In general, chromosome aberrations are generated as a result of meiotic abnormalities, which could be induced, amongst others, by the rapid changes in the occurrence of the repetitive sequences (Ozkan et al., 2001). In our study, we observed several chromosome translocations in F_2_ plants caused by intergenomic chromosome associations during meiosis of PMCs of F_1_ plants. However, the karyotype analysis of F_1_ plants revealed that six plants carried cryptic intergenomic chromosome translocations involving chromosome segments from sub-genomes of both parental forms. It could be hypothesized, that rapid genome changes, triggered by the forcible cross-hybridization, resulted in random chromosome fragmentation and non-homologous end joining. DNA double-strand breaks (DSBs) may result from endogenous and exogenous DNA damage agents or mutagenesis (Friedberg et al., 2004). It was reported that cellular defects caused by the irregularities in mitosis can promote chromosome mis-segregation and aneuploidy. Mitotic failures can result in the DNA damage and chromosome breaks, as well (Ganem and Pellman, 2012). Moreover, abnormal mitosis associated with the DNA damage is reported to have potential impact on tumorigenesis in mammalians. We hypothesized, the similar process of random chromosome fragmentation and non-homologous chromosome end joining occurred at mitosis of cells in the root meristems of F_1_ plants and resulted in a formation of reciprocal translocations (i.e., 1BS–1BL.5 ML and 5MS–5ML.1BL). Moreover, this hypothesis could be endorsed by the heterogeneity of particular chromosome translocations found in different cells of single root meristem. It can be suspected that chromosome fragmentation in this case could be caused by cellular stress (Stevens et al., 2011), however, at the moment we do not have evidence to confirm those statements.

In summary, this study shed some light on the pivotal-differential evolutionary process of speciation. Our results showed that the induction of the intergenomic chromosome translocations in pivotal-differential allopolyploids is a complicated and complex process. There are many genetic and epigenetic factors that could have an influence on this mechanism. We characterized the chromosome translocations and breakpoints and suspected that their localization can be related to the chromosome loci of some similar motifs of repetitive DNA sequences. Our observations revealed that the pivotal genome is crucial for the fertility of F_1_ hybrids, however, chromosomes of the pivotal genome can be also involved in the intergeneric recombination.

## Author contributions

Conceived and designed the project and experiments: MK. Generated the plant material: MK, JB, and HW. Performed the FISH/GISH experiments: MK, JM, MM. Performed the immunodetection experiments: MK. Performed the clone DNA sequences comparison: MM. Analyzed the data: MK. Wrote the paper: MK.

### Conflict of interest statement

The authors declare that the research was conducted in the absence of any commercial or financial relationships that could be construed as a potential conflict of interest.
